# An Integrated Systematic Analysis and the Clinical Significance of Hepcidin in Common Malignancies of the Male Genitourinary System

**DOI:** 10.3389/fgene.2022.771344

**Published:** 2022-05-12

**Authors:** Xiaogang Wang, Qianqian Shi, Pengfeng Gong, Cuixing Zhou, Yunjie Cao

**Affiliations:** Department of Urology, The Third Affiliated Hospital of Soochow University, Changzhou, China

**Keywords:** hepcidin, cancer, male genitourinary system, TCGA, prognosis, immune cell infiltration

## Abstract

Tumors of the male genitourinary system are of great concern to the health of men worldwide. Although emerging experiment-based evidence indicates an association between hepcidin and such cancers, an integrated analysis is still lacking. For this reason, in this study, we determined the underlying oncogenic functions of hepcidin in common male genitourinary system tumors, including bladder urothelial carcinoma (BLCA), kidney chromophobe (KICH), kidney renal clear cell carcinoma (KIRC), kidney renal papillary cell carcinoma (KIRP), prostate adenocarcinoma (PRAD), and testicular germ cell tumors (TGCT) according to the data from The Cancer Genome Atlas. We found that hepcidin was highly expressed in kidney and testicular cancers. Meanwhile, the expression level of hepcidin was distinctly associated with the prognosis and immune cell infiltration in male patients with certain genitourinary system cancers, especially in KIRC. Elevated hepcidin levels also present as a risk factor in male genitourinary system tumors. Moreover, enrichment analyses revealed that some of the principal associated signaling pathways involving hepcidin and its related genes are identified as tumorigenesis-related. Immunofluorescence staining confirmed the conclusion of our immune infiltration analysis in KIRC tissue. In this study, for the first time, we provided evidence for the oncogenic function of hepcidin in different types of male genitourinary system tumors.

## Introduction

The male genitourinary system includes the kidney, ureter, bladder, urethra, testis, prostate, penis, and other organs. Cancers of these organs are common, among which prostate and bladder tumors rank among the top 10 most common tumors in men globally (Ferlay et al., 2021; Islami et al., 2021), due to their long disease cycle and sometimes high medical resource consumption. In addition, the incidence of kidney tumors is also a branch of male genitourinary system tumors that have developed rapidly in recent years, while testicular tumors are relatively rare (Siegel et al., 2021). For some male-specific tumors, continuous enhancement of diagnosis and treatment can significantly improve the survival time and quality of life of patients.

To enhance diagnosis and treatment, integrated analyses of gene expression potentially related to tumorigenesis, correlation assessment of target genes with the prognosis of the diseases, and an investigation of the potential molecular pathways involved in tumorigenesis are of critical importance. The open-access database The Cancer Genome Atlas (TCGA) provides functional genomics data related to tumorigenesis of different types of cancers (Tomczak et al., 2015; Sanchez-Vega et al., 2018; Linehan and Ricketts, 2019), allowing these integrated analyses to be conducted.

One potential compound of interest for this type of analysis is hepcidin (hepcidin antimicrobial peptide, also known as HAMP). Hepcidin is a hormone that was discovered in 1998 and has been successfully isolated from blood plasma and urine to obtain an antibacterial peptide (Leong and Lönnerdal, 2004). It is mainly synthesized in liver cells. It has also been found to be synthesized in the kidneys, the heart, adipose tissue, the spinal cord, and other tissues but only at a very low level (Kroot et al., 2011). The human hepcidin gene is a single-copy gene located on the long arm of chromosome 19 (19Q13). An initial study found that hepcidin was closely related to iron metabolism (Pigeon et al., 2001). Further studies found that hepcidin not only plays an important role in iron metabolism but also closely related to inflammation and tumorigenesis (Ganz, 2003; De Domenico and Kaplan, 2009; Arosio, 2014; Mleczko-Sanecka et al., 2014). Studies have shown that iron promotes the proliferation and metastasis of tumor cells (Mittler et al., 2019) and plays an important role in the occurrence and development of tumors. The expression of hepcidin is significantly upregulated in patients with malignant tumors, and it has been found that tumors that synthesize hepcidin can reduce the expression of membrane iron transporters in an autocrine manner, thereby limiting iron discharge from tumor cells and promoting tumor growth by increasing iron content in tumor cells (Sacco et al., 2021). The mechanism of iron acquisition and retention is thus enhanced in cancer cells (Bordini et al., 2020), and the expression of genes regulating iron metabolism can predict the prognosis of patients with cancer (Wu et al., 2014; Hsu et al., 2020). This suggests that iron-regulating proteins such as hepcidin can be inhibited to expel iron from tumors and that depletion of iron from cancer cells might inhibit tumor growth. Therefore, regulating iron metabolism can be used as an antitumor strategy (Hsu et al., 2020; Lelièvre et al., 2020). Hepcidin has also been previously reported to play an important role in digestive system tumors, including colorectal cancer (Sornjai et al., 2020; Colorectal Cancer Cells Ectopically Express Hepcidin to Sequester Iron, 2021; Schwartz et al., 2021), gastric cancer (Jakszyn et al., 2017), pancreatic cancer (Toshiyama et al., 2018), and hepatocellular carcinoma (Abd Elmonem et al., 2009; Udali et al., 2018; Wang et al., 2019), as well as in thoracic cancers such as breast cancer (Blanchette-Farra et al., 2018; El-Mahdy et al., 2020; Jerzak et al., 2020; Shibabaw et al., 2020; Blindar et al., 2021) and lung cancer (Chen et al., 2014; Fan et al., 2021). Collectively, hepcidin appears to play an important role in cancer.

In this study, an integrated analysis of hepcidin in common male genitourinary system tumors was conducted using data mainly from TCGA project. For a better understanding of the involvement of hepcidin in the underlying mechanism of the tumorigenesis of different types of common male genitourinary system tumors, we studied several factors in hepcidin such as gene alteration, DNA methylation, association with immune infiltration, and clinical significance. These would be helpful for better understanding the value of hepcidin in male genitourinary system tumors.

## Materials and Methods

### Gene Expression Analysis

Hepcidin expression levels in various types and subtypes of cancers in TCGA project and healthy controls were obtained from the database of Tumor IMmune Estimation Resource version 2 (TIMER2, timer.cistrome.org) (Li et al., 2017; Li et al., 2020). The types of common male genitourinary system tumors that we focused on in this study were as follows: bladder urothelial carcinoma (BLCA), kidney chromophobe (KICH), kidney renal clear cell carcinoma (KIRC), kidney renal papillary cell carcinoma (KIRP), prostate adenocarcinoma (PRAD), and testicular germ cell tumors (TGCT). For expression analysis, box plots of Gene Expression Profiling Interactive Analysis version 2 (GEPIA2, gepia2. cancer-pku.cn) were applied for types of cancer with limited information of healthy control (Tang et al., 2019). Also, hepcidin expression in different stages of cancer was summarized as in violin plots using GEPIA2. The transformed transcriptional levels were calculated as log2 of transcripts per million (TPM) +1 and were used in box and violin plots.

The protein-level analysis of the data from the Clinical Proteomic Tumor Analysis Consortium (CPTAC) (Edwards et al., 2015) was conducted using the UALCAN portal (ualcan.path.uab.edu/analysis-prot.html) (Chandrashekar et al., 2017). We explored the expression level of hepcidin between primary tumor and normal tissues, respectively. The correlation between protein expression of hepcidin and patients’ age, gender, disease stage, tumor histology, and body weight was further analyzed. The available datasets were the data of KIRC.

### Survival and Prognosis Analysis

Both overall survival (OS) and disease-free survival (DFS) of hepcidin in various TCGA cancers were obtained using the survival map section of GEPIA2. The threshold for different expression levels was set as high expression (50%) and low expression (50%). A hypothesis test was carried out using the log-rank test. Pooled analysis (univariate Cox regression) of OS, disease-free interval (DFI), progression-free interval (PFI), and disease-specific survival (DSS) was conducted across male genitourinary system tumors. The forest plot was drawn without hazard ratio (HR) merging. Then, the receiver operator characteristic curve (ROC) was performed to determine whether the expression of hepcidin could exactly predict the 1-, 3-, and 5-year survival (OS) of patients in selected types of tumors from TCGA database. The best cutoff value for the expression of hepcidin was calculated. Patients of each type of tumor studied were divided into the high-expression group and low-expression group by using the best cutoff value. Survival analysis was used to validate the OS between groups that were stratified by cutoff value.

### Genetic Alteration and DNA Methylation Analysis

The genetic alteration features of hepcidin were analyzed using TCGA pan-cancer studies in cBioPortal (cbioportal.org) (Gao et al., 2013). Alteration frequencies, mutation types, and copy number alterations in the HAMP gene in selected types of TCGA cancers were analyzed by the cancer types summary. Mutation sites and 3D structures of hepcidin were also analyzed and shown in schematic illustration. The differences in hepcidin genetic alteration-associated OS, DFS, and progression-free survival (PFS) were analyzed. Kaplan–Meier (KM) plots with *p*-values obtained from log-rank tests were produced. Association between different DNA methylation probes and expression of hepcidin was carried out by MEXPRESS (mexpress.be) (Koch et al., 2015; Koch et al., 2019). The beta value of each sample was obtained. The *p*-value (adjusted using the Benjamini–Hochberg procedure) and Pearson correlation coefficient (R) value were obtained. MethSurv (Modhukur et al., 2018) (https://biit.cs.ut.ee/methsurv/) and SurvivalMeth (Zhang et al., 2021) (http://bio-bigdata.hrbmu.edu.cn/survivalmeth/) were used to measure the prognostic significance of DNA methylation of hepcidin in patients with male genitourinary system tumors.

### Immune Infiltration Analysis

The associations between hepcidin levels and infiltrations of CD8^+^ T cells and cancer-associated fibroblasts in selected types of TCGA cancers were analyzed by the Immune-Gene section of TIMER2. For estimating the immune infiltrations, algorithms including TIMER, CIBERSORT, CIBERSORT-abs, quanTIseq, XCELL, MCPCOUNTER, and EPIC were used. Both the *p* value and partial correlation (*r*) value were generated using purity-adjusted Spearman’s rank correlation test. All results were displayed in heatmaps and scatter plots.

### Cancer Immune Analysis

The potential associations between the hepcidin level and tumor mutational burden (TMB), microsatellite instability (MSI), checkpoint expressions, immune cell score (MCPcounter), and immune response pathways in malignancy of the male genitourinary system were derived from Sangerbox (sangerbox.com/Tool) based on TCGA database. Spearman’s rank correlation tests were carried out, and the *p*-value and partial correlation (*r*) value were obtained. Meanwhile, the assessments of OS and relapse-free survival (RFS) were carried out using the interactive operation interface of the KM plotter (kmplot.com) using different data extracted from TCGA database to examine the correlation between patients’ prognoses and immune cell infiltration in tumor tissue. Multiple cellular contents were included in prognostic analysis such as enrichment of B cells, CD4^+^ memory T cells, CD8^+^ T cells, macrophages, mesenchymal stem cells, natural killer T cells, regulatory T cells, type 1 T-helper cells, type 2 T-helper cells. Median was used in the analysis of associations in BLCA, KIRC, KIRP, and TGCT. The HR, 95% confidence intervals, and log-rank *p*-value were computed, and the Kaplan–Meier survival plots were generated.

### Hepcidin**-**Related Gene Enrichment Analysis

The STRING website (string-db.org) was used for the hepcidin-related gene enrichment analysis. The following parameters were used for the analysis: organism, “Homo sapiens”; minimum required interaction score, “low confidence (0.150)”; meaning of network edges, “evidence”; max number of interactors to show, “no more than 50 interactors in first shell”; and active interaction sources, “experiments”. Proteins that were able to bind or interact with hepcidin were generated according to published data of experimentally confirmed data. Meanwhile, the top 100 genes associated with hepcidin were obtained by the similar gene detection section of GEPIA2 according to the data of selected cancer and healthy controls. The correlation analysis was also performed for the Pearson correlation analysis of hepcidin and potential hepcidin-related genes. The log2 of TPM was used for the analysis of dot plots. The *p* value and r value were calculated. Also, the heatmap of related genes was generated by the Gene-Corr section of TIMER2, with r and *p* values calculated by purity-adjusted Spearman’s rank correlation tests. Intersection analyses were conducted for the comparison of hepcidin-related genes using Jvenn (Bardou et al., 2014). The Kyoto Encyclopedia of Genes and Genomes (KEGG) pathway analysis was conducted using combined results obtained from previous analyses. The list of potential hepcidin-related genes was analyzed by the Database for Annotation, Visualization, and Integrated Discovery (DAVID) (Dennis et al., 2003). The enriched signaling pathways were listed in tidyr (cran.rproject org/web/packages/tidyr) and ggplot2 (cran.r-project.org/web/packages/ggplot2) of the R package. The cluster profiler R package (http://www.bioconductor. org/packages/release/bioc/html/clusterProfiler) was conducted for gene ontology (GO) enrichment analysis. Results of biological processes, cellular components, and molecular functions were plotted as cnetplots. R language software [R-4.0.4, 64-bit] (www.r-project.org) was used. Two-tailed analysis (*p* < 0.05) was set as statistical significance.

### Validation in Clinical Samples

The studies involving human participants were reviewed and approved by the Ethics Committee of The Third Affiliated Hospital of Soochow University. The formalin-fixed paraffin-embedded (FFPE) KIRC tissues (N = 10) were obtained from the tissue bank of The Third Affiliated Hospital of Soochow University. The patients had provided their written informed consent when their tissues were stored in the tissue bank. Thus, it is not necessary to re-obtain written informed consent for this study. The FFPE KIRC tissues were used to verify the correlation between the expression of hepcidin and checkpoints in KIRC tissue. Immunofluorescent (IF) staining was performed in FFPE KIRC tissues. Common associated immune checkpoint molecules in male genitourinary system tumors such as CD28, CD48, LGALS9, CTLA4, and PDCD1 were selected for verification (for each aforementioned checkpoint molecular, one FFPE KIRC tissue for testing and another FFPE KIRC tissue for validating). Briefly, the sections were incubated with hepcidin (ab190775, Abcam, MA, United States, 1/100 dilution), CD28 (ab185759, Abcam, MA, United States, 2 μg/ml), CD48 (ab185759, Abcam, MA, United States, 2 μg/ml), LGALS9 (ab153673, Abcam, MA, United States, 5 μg/ml), CTLA4 (ab19792, Abcam, MA, United States, 1/200 dilution), and PDCD1 (ab52587, Abcam, MA, United States, 1/50 dilution) at 4°C overnight and then incubated with Alexa^®^488-conjugated goat anti-rabbit secondary antibody or Alexa^®^576-conjugated goat anti-mouse secondary antibody (Thermo Fisher, CA, United States). The nuclear stain Hoechst 34580 (5 μg/ml; Molecular Probes, Thermo Fisher, CA, United States) was added prior to final washes after the incubation of the secondary antibody. Finally, the sections were dehydrated, cleared, and mounted using a Zeiss confocal microscope. The resulting area and cell measurements were quantified using ImageJ software analysis.

## Results

### Gene Expression Analysis

**FIGURE 1:**
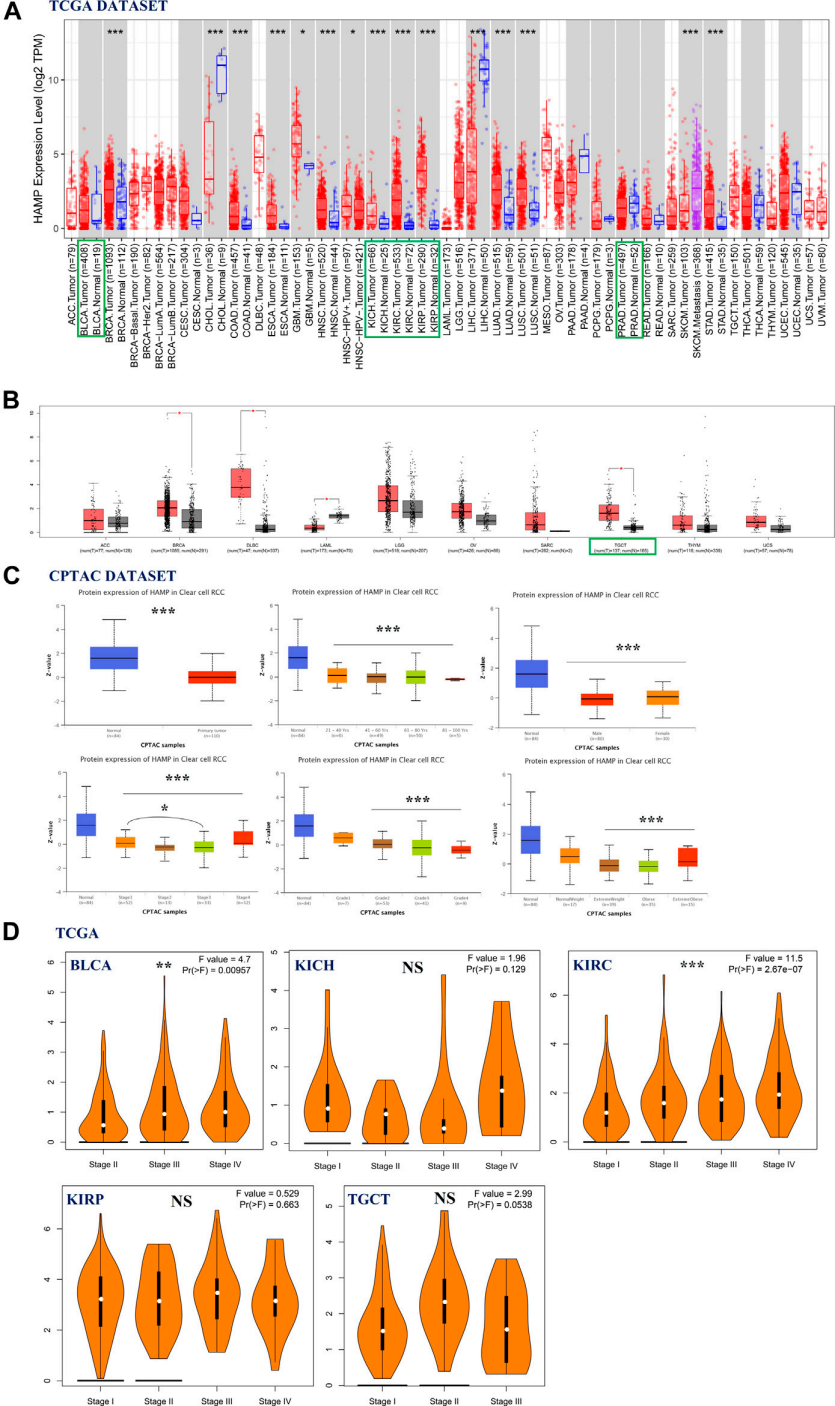
Association of hepcidin levels with clinical parameters in male genitourinary system tumors. **(A,B)** Hepcidin expression in various types or subtypes of cancers and normal tissues as analyzed by TIMER2 and GEPIA2. **(C)** Protein levels of hepcidin in kidney renal clear cell carcinoma (KIRC) tissue and normal tissue combining various clinical parameters as analyzed by CPTAC. **(D)** Using The Cancer Genome Atlas (TCGA) data; hepcidin expression in different pathological stages of several male genitourinary system tumors. *p < 0.05; **p < 0.01; ***p < 0.001].

Hepcidin mRNA expression in common male genitourinary system tumors from TCGA—including BLCA, KICH, KIRC, KIRP, PRAD, and TGCT—was analyzed using TIMER2 and GEPIA2 (Figure 1A, B). Compared to the corresponding healthy controls, elevated levels of hepcidin were found in kidney and testicular tumors: KICH, KIRC, KIRP, and TGCT (*p* < 0.001). For KIRC samples, this trend appears to reverse the trend seen in the protein levels.

Next, based on searches of the CPTAC database, we then compared the differences in total hepcidin protein levels between normal tissues and primary tumor tissues. Only the proteome data from patients with KIRC were analyzed. Interestingly, normal tissue expressed more of the protein than primary tumor tissues. This difference was also confirmed in subgroup analyses, controlling for patients’ age, gender, disease stage, tumor histology, and body weight. For patients with KIRC, there was no significant difference in hepcidin protein expression among different age-groups (21–40; 41–60; 61–80; 81–100 years). Similar results were confirmed in gender groups (male vs. female) and tumor grade groups (grade 1, 2, 3, and 4). It was found that patients with stage 1 cancer had higher hepcidin levels than patients with stage 3 cancer. Patients with normal weight expressed more hepcidin protein than those who were overweight (Figure 1C). The “Pathological Stage Plot” module of GEPIA2 was used to observe the correlation between hepcidin expression and the pathological stages of cancers. Hepcidin was found to be significantly correlated with the stages of BLCA and KIRC among the selected tumors. The mRNA expression of hepcidin is higher in patients with stages III and IV BLCA than those with stage II BLCA, while in KIRC, the mRNA expression of hepcidin increased with the advance of tumor stage. These differences were statistically significant (Figure 1D, *p* < 0.01).

### Survival Analysis

**FIGURE 2:**
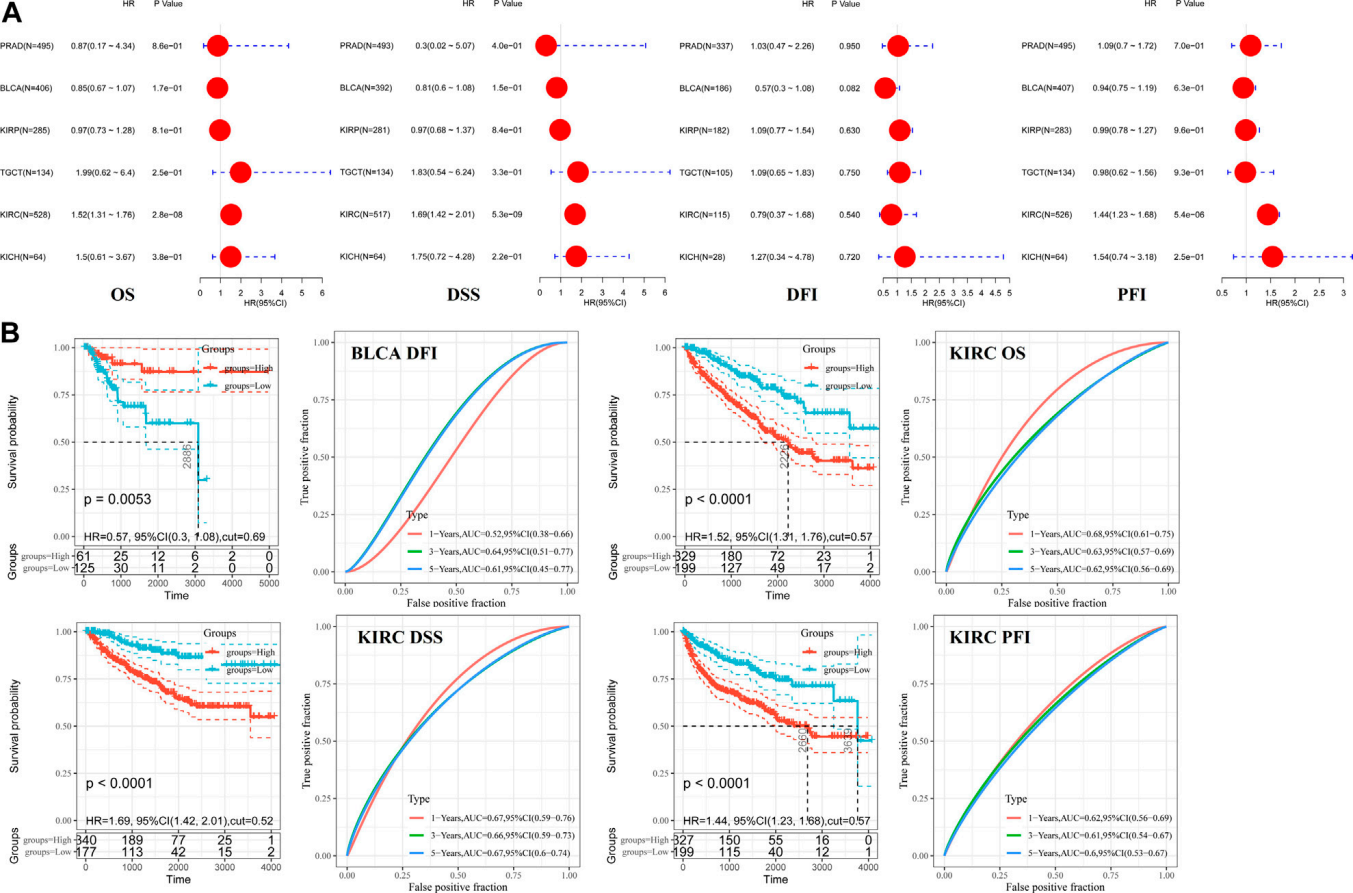
Correlations between hepcidin levels and prognostic survival markers of male genitourinary system tumors. **(A)** Overall survival (OS) and disease-free survival (DFS) related to hepcidin levels in TCGA project as analyzed by GEPIA2. **(B)** Pooled analysis between hepcidin expression and OS, disease-free interval (DFI), disease-specific survival (DSS), and progression-free interval (PFI) were conducted across male genitourinary system tumors using data from TCGA. **(C)** Receiver-operator characteristic curve (ROC) analysis was performed to determine the prediction efficiency of hepcidin for the 1-, 3-, and 5-year OS, DFI, PFI, and DSS of patients with male genitourinary system tumors.

Pooled analyses between hepcidin expression and patient OS, DFI, DSS, and PFI were conducted across the male genitourinary system tumors for which data were available in TCGA. Expression of hepcidin was an independent prognostic factor for OS, DSS, and PFI in KIRC (HR = 1.52, 95% confidence interval (95% CI): 1.31–1.76, *p* < 0.001; HR = 1.69, 95% CI: 1.42–2.01, *p* < 0.001; and HR = 1.44, 95% CI: 1.23–1.68, *p* < 0.001) but not for any clinical outcomes in any other selected tumors (Figure 2A; Table 1 **)**.

**TABLE 1:** Pooled univariate analysis of hepcidin in male genitourinary system tumors.

Cancer type/clinical outcome	p-value	HR	95%CI_lower	95% CI_upper	Sample size
OS					
PRAD	0.863964841	0.868889614	0.174074477	4.337046847	495
BLCA	0.171093137	0.849424749	0.672407444	1.073043451	406
KIRP	0.8072184	0.965938468	0.731243238	1.275960003	285
TGCT	0.249471444	1.987961958	0.617373434	6.401300303	134
KIRC	**2.78E-08**	**1.5181508**	1.310204362	1.759101036	528
KICH	0.378239697	1.496176131	0.610607001	3.666094576	64
DSS					
PRAD	0.401272	0.296541	0.017352	5.067748	493
BLCA	0.151464	0.807471	0.60287	1.08151	392
KIRP	0.843372	0.965186	0.679136	1.371719	281
TGCT	0.334642	1.829823	0.536212	6.244278	134
KIRC	**5.25E-09**	**1.689677**	1.416891	2.014981	517
KICH	0.218102	1.752799	0.717554	4.281636	64
DFI					
PRAD	0.947212	1.026944	0.467438	2.256159	337
BLCA	0.082421	0.569556	0.30176	1.075004	186
KIRP	0.629995	1.089416	0.768899	1.543541	182
TGCT	0.74797	1.089027	0.647308	1.832172	105
KIRC	0.541843	0.791733	0.373871	1.676625	115
KICH	0.724705	1.269019	0.337009	4.778541	28
PFI					
PRAD	0.697405578	1.09393913	0.695727202	1.72007479	495
BLCA	0.626065986	0.944495311	0.750663624	1.188377009	407
KIRP	0.960220937	0.993708742	0.775446469	1.273404552	283
TGCT	0.930807845	0.979591434	0.615042149	1.560217263	134
KIRC	**5.40E-06**	**1.438269766**	1.229784547	1.682099459	526
KICH	0.247387177	1.537149571	0.741892889	3.184865149	64

Bladder urothelial carcinoma (BLCA), kidney chromophobe (KICH), kidney renal clear cell carcinoma (KIRC), kidney renal papillary cell carcinoma (KIRP), prostate adenocarcinoma (PRAD), and testicular germ cell tumors (TGCT). Overall survival (OS), disease-free interval (DFI), progression-free interval (PFI), and disease-specific survival (DSS), hazard ratio (HR), confidence interval (CI). The bold type indicates statistical significance. Bold values are statistically significant (*P*<0.05).

ROC analysis was then performed to determine the prediction efficiency of hepcidin expression for the 1-, 3-, and 5-year prognosis for OS, DFI, PFI, and DSS of patients from TCGA database. The best cutoff value for hepcidin expression was then calculated for each metric. Patients were divided into the high-expression and low-expression groups according to the best cutoff values. Survival analysis was then conducted to validate the prognosis between groups. We defined good prediction efficiency of hepcidin as the area under the curve (AUC) > 0.6, and a *p*-value of survival analysis <0.05. Hepcidin was found to have good prediction efficiency for OS, PFI, and DSS in KIRC (cutoff value 0.57, 1-year AUC 0.68, 3-year AUC 0.63, 5-year AUC 0.62; cutoff value 0.57, 1-year AUC 0.62, 3-year AUC 0.61, 5-year AUC 0.60; and cutoff value 0.52, 1-year AUC 0.67, 3-year AUC 0.66, 5-year AUC 0.67; respectively, all *p* < 0.001) and of DFI in BLCA (cutoff value 0.69, *p* = 0.0053, 3-year AUC 0.64, 5-year AUC 0.61) (Figure 2B). Hepcidin thus shows potential as a prognostic marker of KIRC.

### Genetic Alteration and DNA Methylation Analysis

**FIGURE 3:**
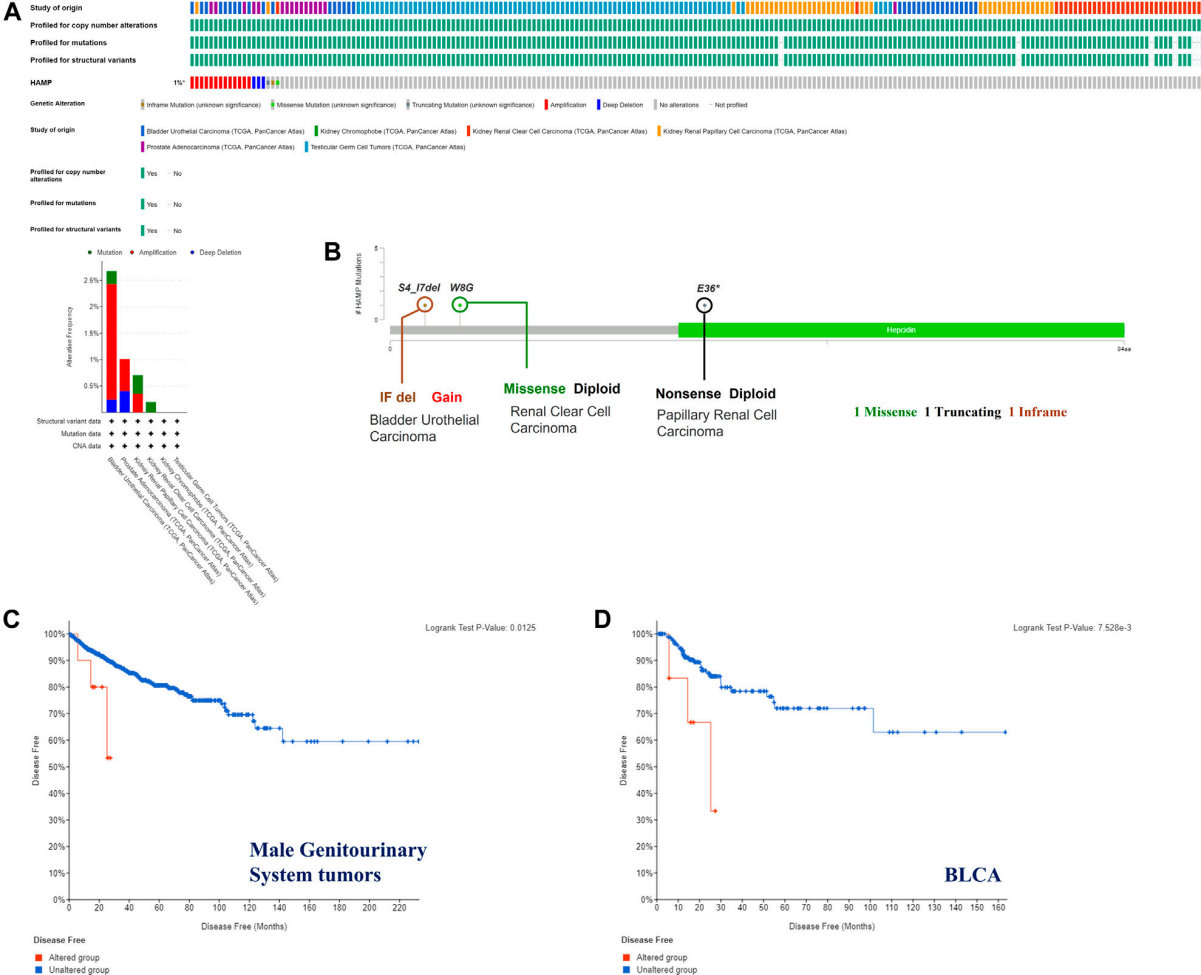
Hepcidin alterations related to male genitourinary system tumors whose data are deposited in TCGA. Hepcidin alteration features analyzed by cBioPortal. **(A)** Alteration pattern, cancer types, and host altered sites **(B)**. There were three major alteration patterns: mutation colored green, amplification colored red, and deep deletion colored blue. Survival analysis (disease-free survival) was performed between the hepcidin alteration group and an unaltered group of **(C)** all male genitourinary system tumors and **(D)** bladder urothelial carcinoma (BLCA) to reveal the clinical significance of hepcidin alteration.

The genetic alterations of the hepcidin gene in common male genitourinary system tumors (BLCA, KICH, KIRC, KIRP, PRCA, and TGCT) were also explored. As a result of mutation frequency summarizing, we found alterations in these tumors are rare, with a general occurrence rate of 1%. Amplification was the most common genetic alteration found in the studied cancer patients, while patients with BLCA had the highest proportion of altered hepcidin genes at about 2.5%. Detailed information on all the genetic alterations found is displayed in Figure 3A. S4_17del, W8G, and E36* mutations were the most frequent site mutations, which were found in one case each of BLCA, KIRC, and KIRP. These mutations were found to induce in-frame, missense, and nonsense mutations, respectively (Figure 3B).

**FIGURE 4:**
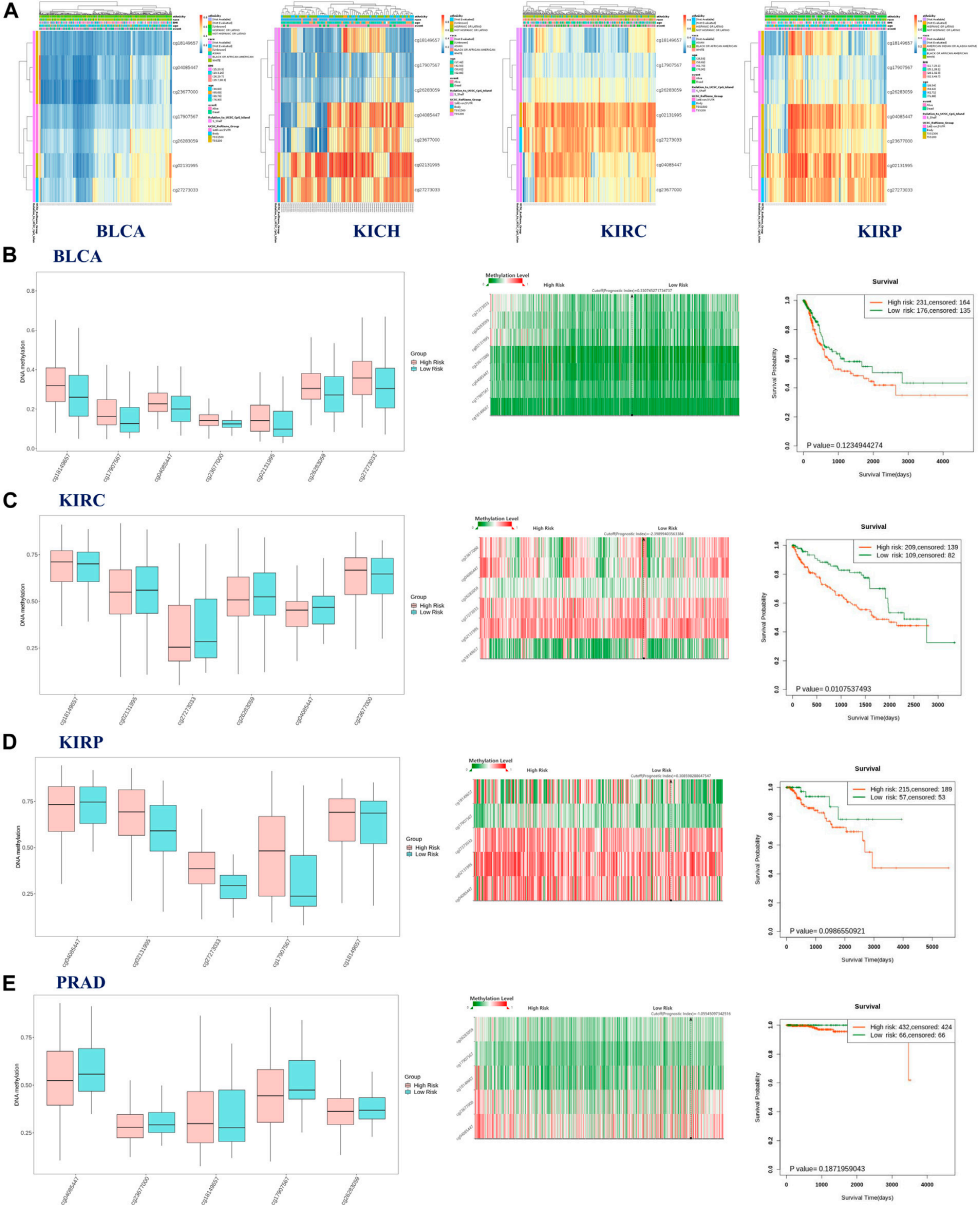
**(A)** Heatmap of DNA methylation level of hepcidin in bladder urothelial carcinoma (BLCA), kidney chromophobe (KICH), kidney renal clear cell carcinoma (KIRC), and kidney renal papillary cell carcinoma (KIRP). The aforementioned results were analyzed by MethSurv. Results of SurvivalMeth showed the correlation between methylation of CpGs in hepcidin and risk stratification and clinical outcomes in BLCA **(B)**, KIRC **(C)**, KIRP **(D)**, and PRAD **(E)**. Left panel: the methylation level of CpGs between high- and low-risk groups; middle panel: the heatmap of single CpG methylation level; right panel: the survival curve between high- and low-risk groups.

The possible correlation between genetic alterations of the hepcidin gene and the prognosis of patients was then carried out. As shown in Figure 3C, the results suggest that the adverse prognosis of DFS in selected tumors was linked to the patients with hepcidin alterations. However, for each type of tumor, an adverse prognosis of DFS was only observed in patients with BLCA (Figure 3D) as the hepcidin alteration proportion of patients with other selected tumors is low. The association of the DNA methylation levels in the hepcidin gene and gene expression in selected tumors from TCGA datasets was analyzed using the visualizer MEXPRESS. The DNA methylation level was found significantly correlated with the hepcidin transcription level at different probes. Data from this analysis are summarized in Table 2 and Supplementary Figure S2. The heatmaps (MethSurv) of DNA methylation of hepcidin in male genitourinary system tumors (BLCA, KICH, KIRC, and KIRP) are shown in Figure 4A. Sites cg02131995 and cg27273033 were found highly methylated in all selected male genitourinary system tumors, and methylations of cg02131995 were associated with prognosis in BLCA and KIRC. Results are summarized in Table 3. Figures 4B–E show different expression patterns of hepcidin between low- and high-risk groups in BLCA (Figure 4B), KIRC (Figure 4C), KIRP (Figure 4D), and PRAD (Figure 4E) that were calculated by SurvivalMeth. The heatmap revealed the CpG methylation level of hepcidin in BLCA (Figure 4B), KIRC (Figure 4C), KIRP (Figure 4D), and PRAD (Figure 4E) which are based on TCGA database. Moreover, statistically prognostic association was only found between the high- and low-risk groups in KIRC patients. The results in the same type of cancer were in keeping with those from MethSurv in general.

**TABLE 2:** Significant correlation of DNA methylation and hepcidin expression at multiple probes.

Variable	*p*-value	Pearson_*r*
BLCA		
cg02131995	2.10705E-06	0.227991588
cg23677000	4.50177E-05	0.196624459
cg04085447	0.000223399	0.178150636
cg17907567	1.40E-08	0.272118082
cg26283059	2.10E-08	0.268768753
cg27273033	2.75749E-06	0.225401479
KIRC		
cg02131995	6.14E-10	−0.32920712
cg18149657	0.002960485	-0.160247851
cg04085447	4.71915E-06	-0.244858437
cg27273033	7.36E-09	-0.307908148
KICH		
cg02131995	0.021181669	-0.285522417
KIRP		
cg02131995	0.00012	−0.2214
cg18149657	0.004127	−0.16598
cg23677000	0.002083	−0.17794
cg04085447	7.66E-06	−0.25658
cg27273033	8.58E-05	−0.22596
PRAD		
cg04085447	0.044002421	-0.087442045
TGCT		
cg18149657	0.035647063	-0.168367271

Bladder urothelial carcinoma (BLCA), kidney chromophobe (KICH), kidney renal clear cell carcinoma (KIRC), kidney renal papillary cell carcinoma (KIRP), prostate adenocarcinoma (PRAD), testicular germ cell tumors (TGCT).

**TABLE 3:** Prognostic value of DNA methylation of hepcidin in patients with male genitourinary system tumors.

	HR	95% confidence interval	*p*-value
BLCA			
cg02131995	**1.446**	**(1.037; 2.017)**	**0.03**
cg27273033	1.249	(0.913; 1.708)	0.16
KICH			
cg02131995	2.39	(0.639; 8.93)	0.2
cg27273033	0.472	(0.127; 1.763)	0.26
KIRC			
cg02131995	**0.518**	**(0.311; 0.863)**	**0.011**
cg27273033	0.748	(0.468; 1.198)	0.23
KIRP			
cg02131995	0.568	(0.277; 1.163)	0.12
cg27273033	0.5	(0.221; 1.131)	0.096

Bladder urothelial carcinoma (BLCA), kidney chromophobe (KICH), kidney renal clear cell carcinoma (KIRC), kidney renal papillary cell carcinoma (KIRP), hazard ratio (HR).

### Immune Infiltration Analysis

**FIGURE 5:**
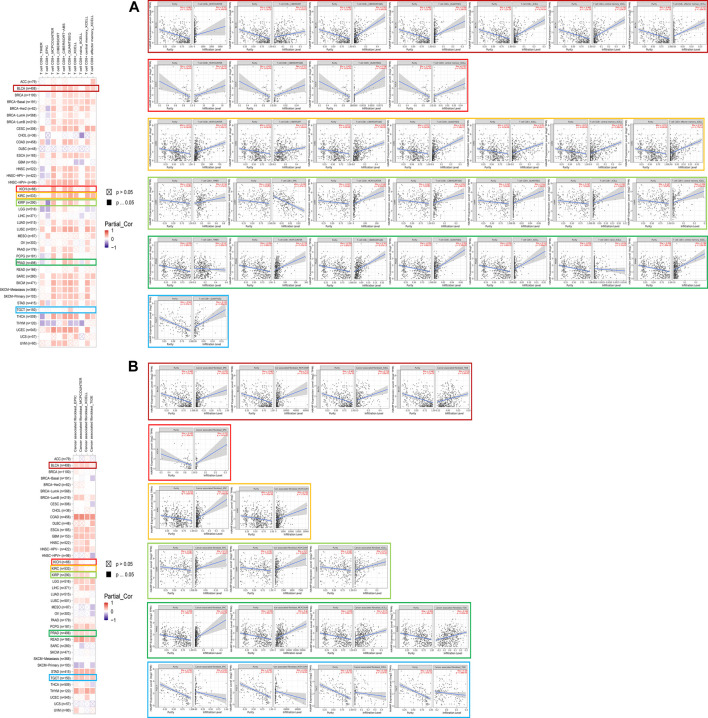
Association between hepcidin and immune infiltration of **(A)** CD8^+^ T cells and **(B)** tumor-associated fibroblast in male genitourinary system tumors in TCGA.

As one of the main features of the tumor microenvironment, immune cell infiltration is tightly related to oncogenesis. Tumor-associated fibroblasts and CD8^+^ T cells located in the stroma of the tumor microenvironment were found to contribute to the regulation of the functions of different tumor-infiltrated immune cells. In this study, the possible correlation between immune cell infiltration and the hepcidin transcription level was analyzed using data from TCGA. In BLCA, KIRC, KIRP, and PRAD, hepcidin was found to be positively correlated with infiltrations of CD8^+^ T cells in most of the tested algorithms. However, in TGCT, most algorithms did not find any correlation between CD8^+^ T-cell infiltration and the level of hepcidin (Figure 5A). Moreover, we found that the hepcidin level was positively correlated with the estimated immune infiltrating value of tumor-associated fibroblasts in BLCA, KIRP, PRAD, and TGCT, while an inverse correlation was found in KICH and KIRC. Every representative correlation plot of the aforementioned cancers as estimated by a single algorithm is shown in Figures 5A, B.

**FIGURE 6:**
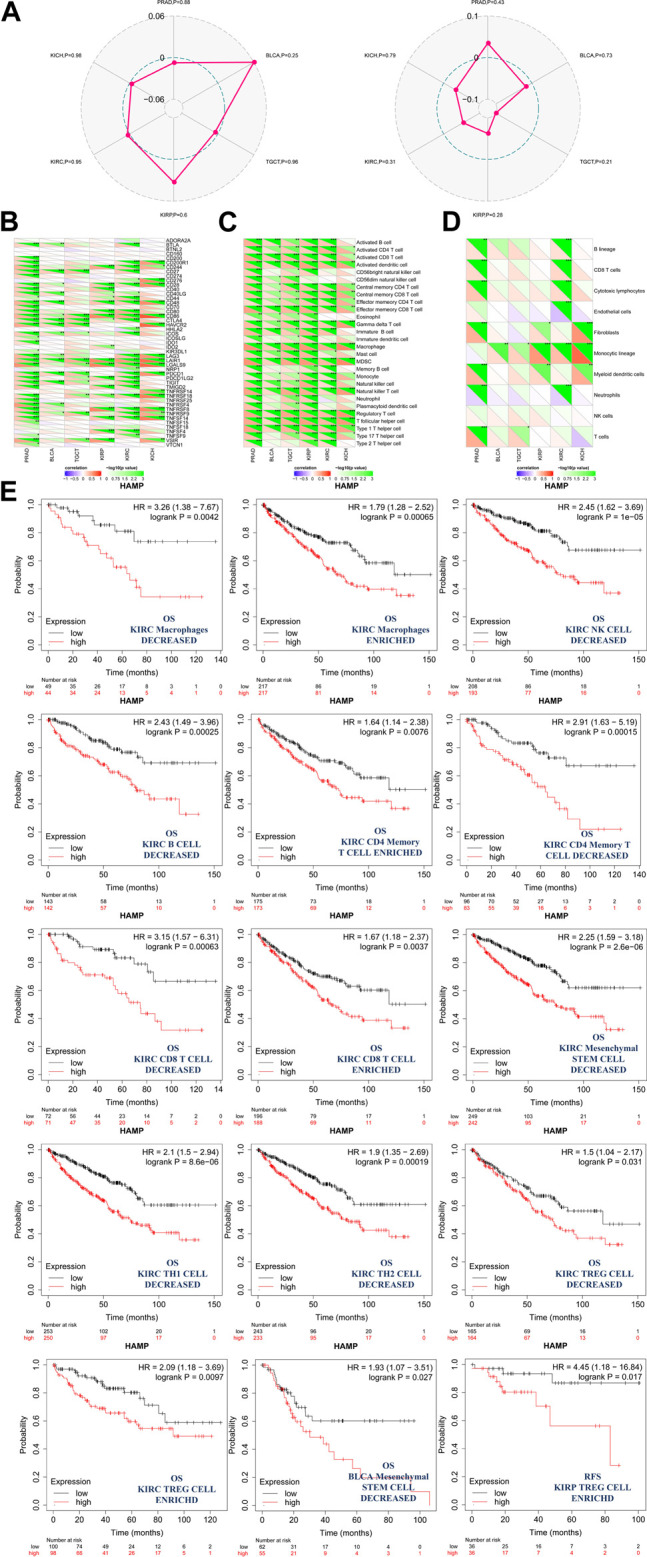
Analysis between hepcidin expression and cancer immunity. **(A)** Correlations between hepcidin levels and tumor mutational burdens (left panel)/microsatellite instabilities (right panel). **(B)** Correlations between expression of hepcidin and immune checkpoints. Correlations between hepcidin levels and **(C)** immune response pathways and **(D)** immune cell score *p < 0.05; **p < 0.01; ***p < 0.001. **(E)** Associations between hepcidin expression and the clinical prognosis of male genitourinary system tumor patients with different immune cell content were analyzed by KM plotter using data extracted from TCGA database. Survival studies related to the expression of hepcidin in bladder urothelial carcinoma (BLCA), kidney renal clear cell carcinoma (KIRC), kidney renal papillary cell carcinoma (KIRP), and testicular germ cell tumors (TGCT) patients were used.

However, there were no correlations found between hepcidin expression and TMB or MSI (Figure 6A). Furthermore, correlation analyses between immune checkpoints and hepcidin revealed positive correlations with most of the checkpoints studied in BLCA, KIRP, KIRC, and KICH (Figure 6B). About half of all checkpoints were found to be positively correlated with hepcidin in BLCA and TGCT. Among all the tested checkpoints, CD28, CD48, CTLA4, LAIR1, LGALS9, PDCD1, TNFRSF18, TNFRSF9, and VSIR were identified to be positively correlated with hepcidin expression in most of the tumors studied.

In the correlation analyses of the immune response pathways, we found positive correlations between hepcidin expression and almost all of the major immune response pathways, with no negative correlations identified. Common malignancies of the male genitourinary system in TCGA were all associated with the aforementioned findings (Figure 6C).

An immune score model was proposed as a prognostic tool for the evaluation of cancers. In this study, we found that the monocytic cell lineage was positively correlated with hepcidin across most of the tumors studied. Hepcidin seemed to be most associated, however, in PRAD and KIRC. In these tumors, lymphocytes and neutrophils were more likely to be correlated with hepcidin expression (Figure 6D). To further study the correlation between patient prognosis and immune cell infiltration in tumor tissues, assessments of patient prognosis were performed using an immune score model. Data extracted from TCGA database provided the cellular content of B cells, CD4^+^ memory T cells, CD8^+^ T cells, macrophages, mesenchymal stem cells, natural killer T cells, regulatory T cells, type-1 T-helper cells, and type-2 T-helper cells in BLCA, KIRC, KIRP, and TGCT tissues. The results revealed that in KIRC, the association between hepcidin and infiltration of immune cells had a significant impact on OS, with high expression indicating worse survival overall. In this model, KIRC patients who had enriched counts of the following were found: macrophages, CD4^+^ memory T cells, CD8^+^ T cells, and regulatory T cells. Meanwhile, there were also patients who had the number of macrophages, natural killer T cells, B cells, CD4^+^ memory T cells, CD8^+^ T cells, mesenchymal stem cells, type 1 T-helper cells, type 2 T-helper cells, and regulatory T cells decreased. All these were found in at least 90 cases, with all *p*-values <0.05. Specifically, macrophage enriched [cases included in analysis (*n*) = 434, HR: 1.79, 95% CI: 1.28–2.52, *p* = 0.00056], macrophage decreased (*n* = 93, HR: 3.26, 95% CI: 1.38–7.67, *p* = 0.0042), natural killer T cells decreased (*n* = 401, HR: 2.45, 95% CI: 1.62–3.69, P = 1e-05), B cells decreased (*n* = 285, HR: 2.43, 95% CI: 1.49–3.96, *p* = 0.00025), CD4^+^ memory T cells enriched (*n* = 348, HR: 1.64, 95% CI: 1.14–2.38, *p* = 0.0076), CD4^+^ memory T cells decreased (*n* = 179, HR: 2.91, 95% CI: 1.63–5.19, *p* = 0.00015), CD8^+^ T cells decreased (*n* = 143, HR: 3.15, 95% CI: 1.57–6.31, *p* = 0.00063), CD8^+^ T cells enriched (*n* = 384, HR: 1.67, 95% CI: 1.18–2.37, *p* = 0.0037), mesenchymal stem cells decreased (*n* = 491, HR: 2.25, 95% CI: 1.59–3.81, *p* = 2.6e-06), type 1 T-helper cells decreased (*n* = 483, HR: 2.1, 95% CI: 1.5–2.94, *p* = 8.6e-06), type 2 T-helper cells decreased (*n* = 476, HR: 1.9, 95% CI: 1.35–2.69, *p* = 0.00019), regulatory T cells decreased (n = 329, HR: 1.5, 95% CI: 1.04–2.17, *p* = 0.031), and regulatory T cells enriched (*n* = 198, HR: 2.09, 95% CI: 1.18–3.69, *p* = 0.0097). KIRC patients all showed increased hepcidin expression and were correlated with poor OS. A high expression of hepcidin was also linked to poor OS in patients with mesenchymal stem cell-decreased BLCA (*n* = 117, HR: 1.93, 95% CI: 1.07–3.51, *p* = 0.027) and to poor RFS in patients with regulatory T-cell-enriched KIRP (*n* = 72, HR: 4.45, 95% CI: 1.18–16.84, *p* = 0.017). The impact of hepcidin expression was thus more pronounced in patients with KIRC, and a higher expression of hepcidin may predict a worse prognosis in patients with KIRC.

### Enrichment Analysis of Hepcidin-Related Partners

**FIGURE 7:**
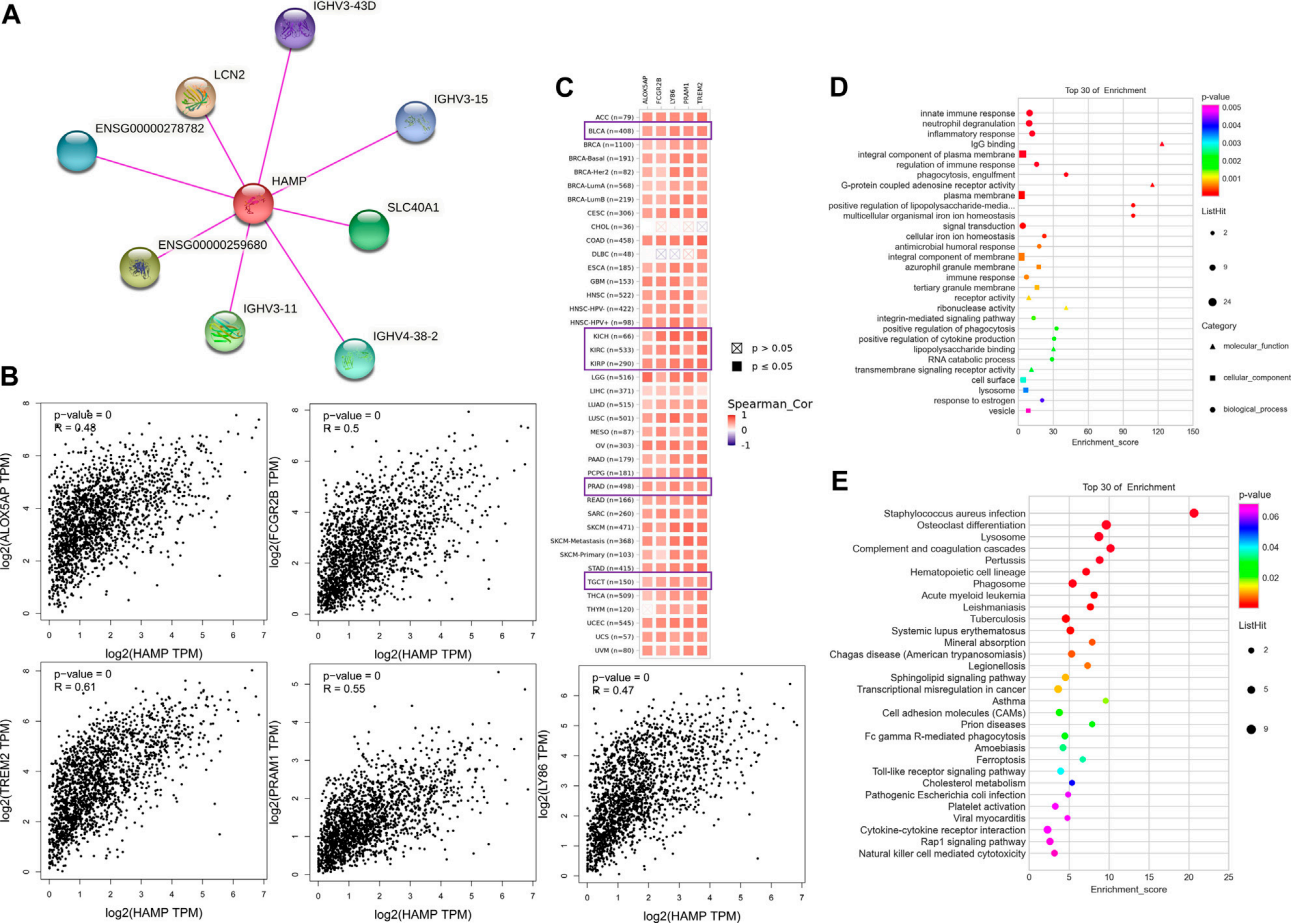
Hepcidin-associated gene enrichment analysis. **(A)** hepcidin-binding proteins analyzed by STRING. **(B)** Correlations between hepcidin and hepcidin-associated genes in TCGA analyzed by GEPIA2. **(C)** Heatmap of corresponding correlation results in different types of cancers. **(D)** Gene ontology (GO) and **(E)** Kyoto encyclopedia of genes and genomes (KEGG) enrichment analysis.

Screening for potential hepcidin-binding proteins and genes associated with hepcidin expression was conducted to investigate the potential mechanisms involved in the effects of hepcidin in oncogenesis. As analyzed using the STRING database, a total of seven hepcidin-binding proteins were extracted. The interaction network between hepcidin and these seven proteins is shown in Figure 7A. GEPIA2 was then used to combine the expression data of the selected tumors from TCGA with the top 100 extracted genes associated with hepcidin levels. Positive correlations were found between the hepcidin gene and ALOX5AP (R = 0.48), LY86 (R = 0.47), PRAM1 (R = 0.55), FCGR2B (R = 0.5), and TREM2 (R = 0.61) (Figure 7B). As demonstrated in the heatmap of these genes (Figure 7C), they were all found to be positively correlated with hepcidin expression in common male genitourinary system tumors.

The KEGG and GO enrichment analyses were then conducted using the combined results of the aforementioned studies. In the GO analysis, the hepcidin-associated genes were found to be involved in the innate immune response, neutrophil degranulation, inflammatory response, IgG binding, integral component of the plasma membrane, regulation of immune response, phagocytosis, engulfment, G-protein-coupled adenosine receptor activity, and plasma membrane (Figure 7D). In the KEGG analysis, the top 10 signaling pathways were as follows: *Staphylococcus aureus* infection, osteoclast differentiation, lysosome, complement and coagulation cascades, pertussis, hematopoietic cell lineage, phagosome, acute myeloid leukemia, and leishmaniasis (Figure 7E). Detailed results are summarized in Supplementary Table S1. Based on the GO and KEGG analyses, the function of hepcidin-associated genes is mainly focused on the innate immunity response. However, we also noticed that several signaling pathways related to oncogenesis have been identified, including transcriptional misregulation in cancer (*p* = 0.012), cell adhesion molecules (CAMs) (*p* = 0.021), and the Toll-like receptor signaling pathway (*p* = 0.012), all of which may contribute to the oncogenesis of cancer (Figure 7E). These might explain why hepcidin-associated immune cells play a critical role in male genitourinary system tumors.

### Validation in Tumor Tissue

**FIGURE 8:**
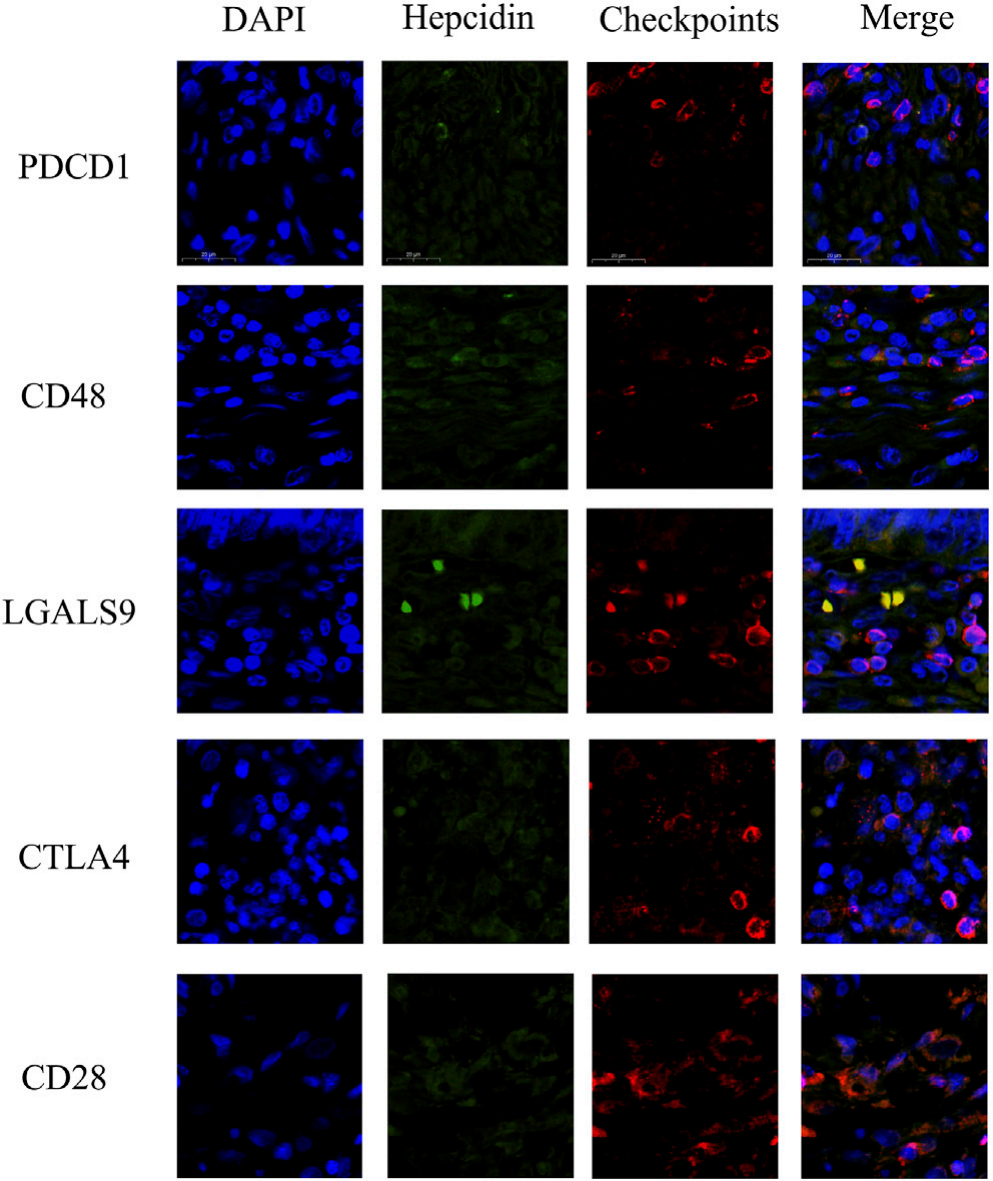
Immunofluorescence staining results showing the co-expression and co-localization between hepcidin and selected immune checkpoints in formalin-fixed paraffin-embedded kidney renal clear cell carcinoma (KIRC) tissue. The verified checkpoint molecules were as follows: CD28, CD48, LGALS9, CTLA4, and PDCD1. These checkpoint molecules were confirmed as the common associated immune checkpoint molecules in male genitourinary system tumors.

To verify the correlation between hepcidin and immune checkpoint molecule in Figures 6B-F,IF staining was carried out in FFPE KIRC tissues. Checkpoint molecules such as CD28, CD48, LGALS9, CTLA4, and PDCD1 were selected for validation. These checkpoint molecules were previously found to be positively correlated with the hepcidin expression in not only KIRC but also other common male genitourinary system tumors through bioinformatics analyses. As shown in Figure 8, the results of IF in FFPE KIRC tissues showed that the expression of hepcidin was weak; meanwhile, all the selected checkpoint molecules expressed less either. This result indicated that the expression of hepcidin and these checkpoint molecules were positively associated in KIRC tissue, in line with the conclusion of bioinformatics analyses.

## Discussion

This study aimed to analyze the functions of hepcidin and provide an overview of its potential role in common male genitourinary system tumors. Previous studies have already revealed the adverse effect of hepcidin in prostate cancer (Tesfay et al., 2015; Wang et al., 2017; Zhao et al., 2018); however, no similar analysis of hepcidin expression in male genitourinary system tumors has been published. The detailed function and molecular signaling pathways other than iron metabolism of hepcidin in male genitourinary system tumors have also not been studied. Therefore, in this study, we provided a comprehensive evaluation of the potential functions of hepcidin in different male genitourinary system tumors using data mainly extracted from TCGA and CPTAC databases combined with an analysis of hepcidin gene expression, genetic alteration, DNA methylation, and gene interactions. We successfully established correlations of these gene features with clinical prognosis. Moreover, validations in KIRC samples were performed to support our conclusion further.

As previously described in many studies, hepcidin was found only highly expressed in the liver, where it is synthesized, with relatively high tissue-specific expression. As revealed by our single-cell resolution analysis, hepcidin was found to be expressed in some macrophages of the kidney and prostate but not in the testis where male genitourinary system tumors usually occur. Hepcidin mRNA however was found highly expressed in both kidney and testis tumors, suggesting the potential role of hepcidin in the development of such cancers. Elevated hepcidin mRNA levels were also found in KICH, KIRC, KIRP, and TGCT. However, this finding was not supported by the protein-level analysis. According to the data extracted from CPTAC, hepcidin protein levels were decreased in KIRC tissues, suggesting that the mRNA did not effectively translate into protein. For eukaryotes, the 3′ untranslated region (UTR) at the 3′ terminal of mRNA is closely related to the efficiency and degradation of mRNA translation. It was believed that AU-rich elements (AREs) on the mRNA could accelerate the degradation of the mRNA. ARE is a typical structure that is consists of tightly regulated genes, such as c-myc, whose half-life is less than 30 min. Caput et al. (1986) found that when mRNA 3′UTR contained an ARE and/or an oligo (U) region, the trend of mRNA instability was enhanced. The AUUUA oligonucleotide sequence and U-rich region in ARE can promote mRNA degradation and reduce mRNA stability (Alberta et al., 1994). Because AREs may inhibit the binding of polymeric (A) sequence and polymeric (A) binding protein, the 3′ terminal of mRNA is easily recognized and degraded by nuclease. Moreover, ARE itself may activate another sequence on the mRNA, making itself a site of specific nuclease attack and thus degradation. Furthermore, the mRNA translation initiation region (translation initiation region, TIR) generally refers to the sequence from upstream -35 to downstream +35 of initiation codon ATG, which contains the binding sites of the initiation codon and ribosome and also includes multiple cis-elements related to translation initiation (Ganoza et al., 1987). A study has shown that the formation of specific secondary structures by partial base pairing in the TIR range can adversely affect translation. A high GC content in this region will lead to the formation of a stable secondary structure of the mRNA transcript product, hinder ribosome sliding, and affect the efficiency of translation (Milon et al., 2008). Therefore, the aforementioned mechanism regarding the mRNA structure instability may be contributed to the low translation level of hepcidin in common male genitourinary system tumors, which may be caused by the tuning of tumor cells and tumor immune microenvironment. Another possibility is that the limited sample size of patients with KIRC (n = 194) in CPTAC may mask the consistency between mRNA and protein levels in patients with this disease. The expression of hepcidin seemed to be less associated with clinical parameters. The only significant association between hepcidin expression and tumor stages was observed between patients with stage 1 and stage 3 cancers.

Survival analyses were conducted using GEPIA2 (Yuzhalin et al., 2018), and the results suggested that in KIRC, the expression of hepcidin was negatively related to OS and DFS, of which high hepcidin expression was related to poor prognosis in KIRC. This finding was confirmed by a pooled analysis of the data from several male genitourinary system tumors. Hepcidin plays a critical role in the prognosis of patients with KIRC, wherein a higher hepcidin expression was related to a worse OS, DSS, and PFI. We further determined if hepcidin is a predictor of male genitourinary system tumors using ROC analysis. As expected, a difference in hepcidin expression predicted 1-, 3-, and 5-year prognosis in patients with KIRC. The cutoff value for which hepcidin becomes a risk factor varies depending on the type of prognosis. In addition to KIRC, hepcidin expression also showed potential in predicting 3- and 5-year prognoses of DFI in BLCA, with a cutoff value of 0.69. A clinical cohort study is needed for further external validation, and the model should be tested against a larger dataset of different tumors to strengthen it further.

Although gene alterations are rarely observed in male genitourinary system tumors, our study showed that genetic alterations were associated with poor survival, especially in BLCA. The DNA methylation level was significantly correlated with the gene transcription level at different probes in male genitourinary system tumors. This observation should be further researched and verified in clinical practice.

As revealed by the data extracted from TCGA, the correlation between hepcidin and immune cell content had a significant impact on the OS in patients with KIRC. Several studies have shown that hepcidin-associated immune cell infiltration is involved in the development of cancers (Shibabaw et al., 2020; Fan et al., 2021), but renal cancer was never mentioned. In this study, the infiltration of most immune cells was linked with elevated hepcidin levels, including those of macrophages, natural killer T cells, B cells, CD4^+^ memory CD8^+^ T cells, mesenchymal stem cells, type 1 T-helper cells, type 2 T-helper cells, and regulatory T cells. Thus, there is potential for hepcidin to be an immune predictor in renal cancer or at least in KIRC.

Bioinformatics has provided us with sufficient information to justify looking into hepcidin further; however, such findings are only predictions that must be verified further through other methods. In this study, we performed an IF assay on FFPE KIRC tissue to verify the finding regarding immune checkpoints. In the bioinformatics analysis, CD28, CD48, CTLA4, LAIR1, LGALS9, PDCD1, TNFRSF18, TNFRSF9, and VSIR were identified to be universally associated with hepcidin expression in male genitourinary system tumors. Two well-known molecular checkpoints, CTLA4 and PDCD1, were also selected for validation in this study. The results were in accordance with bioinformatics data from TCGA, which supports the possibility that hepcidin may be a possible immunotherapeutic target.

More studies are necessary to determine the exact role hepcidin plays in the process of tumor development. It is currently unknown whether hepcidin initiates oncogenesis or is upregulated as a result of tumorigenic development. As a regulator of iron metabolism, studies of hepcidin were more concerned with its function in inflammatory and anemic abnormalities, including in tumors (Scimeca and Bonanno, 2018; Wang et al., 2019; Cheng et al., 2020). Therefore, this study also aimed to elucidate whether hepcidin was involved in any tumorigenic pathways. We found no evidence that suggests a correlation between hepcidin and MSI or TMB in male genitourinary system tumors. By combining data from hepcidin-interacting elements and hepcidin transcription-associated genes in male genitourinary system tumors, an enriched pathway analysis was conducted, and the potential effects of cancer-associated signaling pathways, including transcriptional misregulation in cancer, CAMs, and the Toll-like receptor signaling pathway, were identified. Some of our findings were partly mentioned in previous studies (Li et al., 2020), but more *in vitro* and *in vivo* research studies are still needed for further verification.

We believe that iron metabolism regulation is the key mechanism through which hepcidin influences the development of male genitourinary system cancers. For the first time, our findings revealed a link between hepcidin levels and the infiltration of cancer-associated fibroblasts in the BLCA, KIRP, PRAD, and TGCT models. As a result, more analyses are needed, such as transcriptomics or single-cell genome sequencing, to uncover more evidence for the possible link between hepcidin and tumor immunology.

This systemic analysis of hepcidin is the first report of the significance of hepcidin in the clinical prognosis of common male genitourinary system tumors, as evidenced by its correlation with gene alterations, DNA methylation, cancer immune infiltration, and therapeutic response. Our findings provided an integral overview of the effects of hepcidin in common male genitourinary system tumors.

## Data Availability

The original contributions presented in the study are included in the article/Supplementary Material, further inquiries can be directed to the corresponding author.
