# Supplementary material for: An Integrated Systematic Analysis and the Clinical Significance of Hepcidin in Common Malignancies of the Male Genitourinary System
Source: Front Genet. 2022 May 12;13:771344. doi: 10.3389/fgene.2022.771344 (PMC9133565; doi:10.3389/fgene.2022.771344)
Supplement: Supplementary file 1 [file Table1.DOCX]

**Table S1 Summarized positive results of GO and KEGG enrichment analysis of HEPCIDIN and related genes**

| **Description** | **Gene Related** | **P-value** |
| --- | --- | --- |
| **KEGG** |  |  |
| Staphylococcus aureus infection | C3;SELPLG;ITGB2;C1QB;FCGR1A;C1QC;C3AR1;FCGR2B | 3.25E-09 |
| Osteoclast differentiation | LILRA2;TREM2;LILRA4;SPI1;TYROBP;FCGR1A;CSF1R;CYBA;FCGR2B | 2.80E-07 |
| Lysosome | CD63;PLA2G15;CTSZ;LAPTM5;CD68;NAGPA;SLC11A1;PSAP | 2.97E-06 |
| Complement and coagulation cascades | C3;ITGB2;C1QB;VSIG4;C1QC;C3AR1 | 2.36E-05 |
| Pertussis | C3;ITGB2;C1QB;CD14;C1QC | 0.000235 |
| Hematopoietic cell lineage | CD4;CD33;CD14;FCGR1A;CSF1R | 0.000632 |
| Phagosome | C3;ITGB2;CD14;FCGR1A;CYBA;FCGR2B | 0.000755 |
| Acute myeloid leukemia | CSF1R;CD14;FCGR1A;SPI1 | 0.001411 |
| Leishmaniasis | C3;ITGB2;CYBA;FCGR1A | 0.001756 |
| Tuberculosis | FCER1G;C3;ITGB2;CD14;FCGR1A;FCGR2B | 0.001799 |
| Systemic lupus erythematosus | C3;C1QB;FCGR1A;C1QC;CD86 | 0.002692 |
| Mineral absorption | SLC34A2;SLC40A1;FTL | 0.00633 |
| Chagas disease (American trypanosomiasis) | PLCB2;C3;C1QB;C1QC | 0.006596 |
| Legionellosis | C3;ITGB2;CD14 | 0.00781 |
| Sphingolipid signaling pathway | ADORA3;FCER1G;ADORA1;PLCB2 | 0.011299 |
| Transcriptional misregulation in cancer | SPI1;CD14;FCGR1A;CD86;CSF1R | 0.011821 |
| Asthma | RNASE3;FCER1G | 0.018205 |
| Cell adhesion molecules (CAMs) | CD4;CD86;ITGB2;SELPLG | 0.020979 |
| Prion diseases | C1QC;C1QB | 0.026264 |
| Fc gamma R-mediated phagocytosis | ARPC1B;FCGR2B;FCGR1A | 0.029155 |
| Amoebiasis | ITGB2;CD14;PLCB2 | 0.033474 |
| Ferroptosis | SLC40A1;FTL | 0.035498 |
| Toll-like receptor signaling pathway | CD86;CD14;SPP1 | 0.040051 |
| **GO** |  |  |
| innate immune response | LILRA2;FCER1G;TREM2;LILRA4;RNASE3;CD180;LCN2;TNFAIP8L2;TYROBP;C1QC;CD300LB;LY86 | 2.23E-09 |
| neutrophil degranulation | ALDH3B1;CD63;FCER1G;RNASE3;LCN2;LAIR1;CTSZ;TYROBP;RNASE2;NFAM1;CD68;SLC11A1 | 3.34E-09 |
| inflammatory response | ADORA3;CD180;TNFRSF10C;THEMIS2;ADORA1;AIF1;NFAM1;SLC11A1;LY86 | 4.88E-08 |
| IgG binding | FCER1G;FCGR1B;FCGR2B | 1.35E-06 |
| integral component of plasma membrane | LILRA2;SLC34A2;CD63;FCER1G;LILRA4;ADORA3;CD180;TNFRSF10C;SLC40A1;ADORA1;TYROBP;LAPTM5;NAGPA;SLC11A1;DCBLD2 | 7.11E-06 |
| regulation of immune response | TREM2;LAIR1;TYROBP;PILRA;CD300LB | 1.52E-05 |
| phagocytosis, engulfment | TREM2;FCER1G;AIF1 | 4.91E-05 |
| G-protein coupled adenosine receptor activity | ADORA3;ADORA1 | 0.000117 |
| plasma membrane | FCER1G;ADORA3;TREM2;APBB1IP;SLC11A1;ALDH3B1;CD63;TNFRSF12A;TNFRSF10C;FCGR1B;CTSZ;LAIR1;SLC40A1;NFAM1;CD300LB;PARVG;SLC34A2;ADAP2;ADORA1;TYROBP;PILRA;CD68;LY86;FCGR2B | 0.000123 |
| positive regulation of lipopolysaccharide-mediated signaling pathway | CD180;LY86 | 0.00016 |
| multicellular organismal iron ion homeostasis | SLC11A1;SLC40A1 | 0.00016 |
| signal transduction | LILRA2;ADORA3;TNFRSF10C;APBB1IP;UNC5CL;PRAM1;ADORA1;TYROBP;PILRA;NFAM1 | 0.000183 |
| cellular iron ion homeostasis | LCN2;SLC11A1;SLC40A1 | 0.000314 |
| antimicrobial humoral response | LCN2;RNASE3;SLC11A1 | 0.000601 |
| integral component of membrane | UNC5CL;LST1;TREM2;ASPHD1;MS4A6A;TNFRSF12A;FCGR1B;LAIR1;VSIG4;SLC40A1;NFAM1;MS4A4A;CD300LB;PLD4;SLC34A2;SPNS3;GAL3ST4;PILRA;SLC15A3;CD68;NAGPA;FCGR2B | 0.000618 |
| azurophil granule membrane | NFAM1;CD68;CD63 | 0.000656 |
| immune response | TNFRSF10C;FCGR1B;LST1;C1QC;FCGR2B | 0.000657 |
| tertiary granule membrane | LAIR1;SLC11A1;FCER1G | 0.000852 |
| receptor activity | LILRA2;TREM2;CD180;SLC40A1 | 0.000953 |
| ribonuclease activity | RNASE3;RNASE2 | 0.001043 |
| integrin-mediated signaling pathway | PRAM1;FCER1G;TYROBP | 0.001496 |
| positive regulation of phagocytosis | SLC11A1;FCER1G | 0.001635 |
| positive regulation of cytokine production | NFAM1;SLC11A1 | 0.001863 |
| lipopolysaccharide binding | RNASE3;TREM2 | 0.001938 |
| RNA catabolic process | RNASE3;RNASE2 | 0.002106 |
| transmembrane signaling receptor activity | NFAM1;TNFRSF10C;FCGR2B | 0.002294 |
| cell surface | CD63;FCER1G;TNFRSF12A;CTSZ;TYROBP;DCBLD2 | 0.002846 |
| lysosome | LAPTM5;PLA2G15;CTSZ;SLC11A1 | 0.003479 |
| response to estrogen | SLC34A2;ARPC1B | 0.004198 |
| vesicle | ALDH3B1;SLC34A2;SPNS3 | 0.005139 |
| humoral immune response | TREM2;LY86 | 0.006096 |
| endonuclease activity | RNASE3;RNASE2 | 0.006877 |
| apoptotic signaling pathway | TNFRSF10C;ADORA1 | 0.007389 |
| phagocytosis | SLC11A1;ADORA1 | 0.007389 |
| wound healing | SLC11A1;DCBLD2 | 0.008315 |
| extracellular exosome | ALDH3B1;CD63;GAL3ST4;SDSL;RNASE3;PLA2G15;LCN2;LAIR1;CTSZ;VSIG4;PILRA;RNASE2;C1QC;FCGBP;ARPC1B | 0.008524 |
| specific granule lumen | LCN2;CTSZ | 0.010977 |
| ficolin-1-rich granule membrane | SLC11A1;FCER1G | 0.012612 |
| Golgi cisterna membrane | NAGPA;GAL3ST4 | 0.013757 |
| lysosomal membrane | LAPTM5;CD63;SLC15A3;SPNS3 | 0.014284 |
| O-acyltransferase activity | PLA2G15 | 0.017234 |
| inositol 1,3,4,5 tetrakisphosphate binding | ADAP2 | 0.017234 |
| aldehyde dehydrogenase [NAD(P)+] activity | ALDH3B1 | 0.017234 |
| phospholipid binding | PLA2G15;TREM2 | 0.018333 |
| endothelium development | SLC40A1 | 0.020092 |
| immunoglobulin mediated immune response | FCER1G | 0.020092 |
| lymphocyte homeostasis | SLC40A1 | 0.020092 |
| positive regulation of calcium-mediated signaling | TREM2 | 0.020092 |
| positive regulation of integrin-mediated signaling pathway | CD63 | 0.020092 |
| mast cell activation | FCER1G | 0.020092 |
| negative regulation of blood vessel diameter | ADORA1 | 0.020092 |
| dendritic cell differentiation | TREM2 | 0.020092 |
| respiratory burst | SLC11A1 | 0.020092 |
| glycolipid biosynthetic process | GAL3ST4 | 0.020092 |
| intracellular receptor signaling pathway | DCBLD2 | 0.020092 |
| parathyroid gland development | HOXA3 | 0.020092 |
| glycerophospholipid metabolic process | PLA2G15 | 0.020092 |
| macrophage activation involved in immune response | TYROBP | 0.020092 |
| iron ion transmembrane transporter activity | SLC40A1 | 0.020646 |
| 3'-phosphoadenosine 5'-phosphosulfate binding | GAL3ST4 | 0.020646 |
| azurophil granule lumen | RNASE3;RNASE2 | 0.022228 |
| lipid catabolic process | PLD4;ADORA1 | 0.022295 |
| cell-matrix adhesion | CD63;PARVG | 0.023032 |
| integral component of postsynaptic membrane | ADORA1 | 0.023596 |
| defense response to Gram-negative bacterium | RNASE3;SLC11A1 | 0.023779 |
| sodium-dependent phosphate transmembrane transporter activity | SLC34A2 | 0.024046 |
| glutathione peroxidase activity | GPX1 | 0.024046 |
| phospholipase C activity | ADORA1 | 0.024046 |
| negative regulation of cardiac muscle contraction | ADORA1 | 0.024062 |
| regulation of B cell differentiation | NFAM1 | 0.024062 |
| cellular response to low-density lipoprotein particle stimulus | FCER1G | 0.024062 |
| macrophage activation | SLC11A1 | 0.024062 |
| negative regulation of synaptic transmission, GABAergic | ADORA1 | 0.024062 |
| cellular response to lipoteichoic acid | TREM2 | 0.024062 |
| positive regulation of B cell receptor signaling pathway | NFAM1 | 0.024062 |
| sphingosine-1-phosphate signaling pathway | SPNS3 | 0.024062 |
| positive regulation of epidermal growth factor-activated receptor activity | ADORA1 | 0.024062 |
| specific granule membrane | ALDH3B1;LAIR1 | 0.024423 |
| sodium ion binding | SLC34A2 | 0.027435 |
| unconventional myosin complex | MYO1F | 0.027475 |
| positive regulation of T cell migration | AIF1 | 0.028017 |
| phosphate ion transport | SLC34A2 | 0.028017 |
| positive regulation of potassium ion transport | ADORA1 | 0.028017 |
| epithelial tube branching involved in lung morphogenesis | CTSZ | 0.028017 |
| angiotensin maturation | CTSZ | 0.028017 |
| negative regulation of synaptic transmission, glutamatergic | ADORA1 | 0.028017 |
| locomotion | SPNS3 | 0.028017 |
| cellular phosphate ion homeostasis | SLC34A2 | 0.028017 |
| response to lipopolysaccharide | SLC11A1;TNFRSF10C | 0.030105 |
| sodium:phosphate symporter activity | SLC34A2 | 0.030812 |
| phosphate ion binding | SLC34A2 | 0.030812 |
| peptidoglycan binding | TREM2 | 0.030812 |
| cell cortex region | CTSZ | 0.031339 |
| microglial cell activation | AIF1 | 0.031956 |
| complement activation | C1QC | 0.031956 |
| negative regulation of toll-like receptor 4 signaling pathway | LILRA2 | 0.031956 |
| negative regulation of lipopolysaccharide-mediated signaling pathway | LILRA2 | 0.031956 |
| proteoglycan biosynthetic process | GAL3ST4 | 0.031956 |
| ruffle assembly | AIF1 | 0.031956 |
| iron ion transport | SLC11A1 | 0.031956 |
| negative regulation of lipid catabolic process | ADORA1 | 0.031956 |
| secretory granule membrane | ALDH3B1;TYROBP | 0.03234 |
| Fc-epsilon receptor signaling pathway | FCER1G;LILRA4 | 0.032631 |
| HMG box domain binding | HOXA3 | 0.034178 |
| endosome lumen | CD63 | 0.035188 |
| negative regulation of T cell activation | TNFAIP8L2 | 0.035879 |
| endosome to melanosome transport | CD63 | 0.035879 |
| oligosaccharide metabolic process | GAL3ST4 | 0.035879 |
| innate immune response in mucosa | RNASE3 | 0.035879 |
| cellular response to organic substance | CD68 | 0.035879 |
| positive regulation of monocyte chemotaxis | AIF1 | 0.035879 |
| positive regulation of blood pressure | ADORA1 | 0.035879 |
| aldehyde dehydrogenase (NAD) activity | ALDH3B1 | 0.037532 |
| small molecule binding | LCN2 | 0.037532 |
| extracellular space | CD63;RNASE3;PLA2G15;LCN2;CTSZ;C1QC;LY86 | 0.038755 |
| multivesicular body membrane | CD63 | 0.039021 |
| mRNA stabilization | SLC11A1 | 0.039787 |
| manganese ion transport | SLC11A1 | 0.039787 |
| regulation of respiratory gaseous exchange by neurological system process | ADORA1 | 0.039787 |
| fatty acid catabolic process | PLA2G15 | 0.039787 |
| ethanol catabolic process | ALDH3B1 | 0.039787 |
| defense response to protozoan | SLC11A1 | 0.039787 |
| antigen binding | LILRA2 | 0.040875 |
| tumor necrosis factor-activated receptor activity | TNFRSF10C | 0.040875 |
| actin binding | MYO1F;PARVG;ARPC1B | 0.042371 |
| Arp2/3 protein complex | ARPC1B | 0.04284 |
| axolemma | ADORA1 | 0.04284 |
| positive regulation of extrinsic apoptotic signaling pathway | TNFRSF12A | 0.043679 |
| cellular response to extracellular stimulus | AIF1 | 0.043679 |
| temperature homeostasis | ADORA1 | 0.043679 |
| detection of temperature stimulus involved in sensory perception of pain | ADORA1 | 0.043679 |
| positive regulation of interleukin-10 production | FCER1G | 0.043679 |
| negative regulation of smooth muscle cell proliferation | AIF1 | 0.043679 |
| response to electrical stimulus | AIF1 | 0.043679 |
| complement activation, classical pathway | C1QC | 0.043679 |
| MHC class I protein binding | PILRA | 0.044207 |
| ion transport | LCN2;SLC15A3 | 0.046428 |
| carboxypeptidase activity | CTSZ | 0.047527 |
| vacuolar acidification | SLC11A1 | 0.047556 |
| sulfur compound metabolic process | GAL3ST4 | 0.047556 |
| thyroid gland development | HOXA3 | 0.047556 |
| protein targeting to lysosome | NAGPA | 0.047556 |

**Abbreviation:** Gene ontology (GO), Kyoto encyclopedia of genes and genomes (KEGG)
