# Supplementary figures and images for: An Integrated Systematic Analysis and the Clinical Significance of Hepcidin in Common Malignancies of the Male Genitourinary System
Source: Front Genet. 2022 May 12;13:771344. doi: 10.3389/fgene.2022.771344 (PMC9133565; doi:10.3389/fgene.2022.771344)

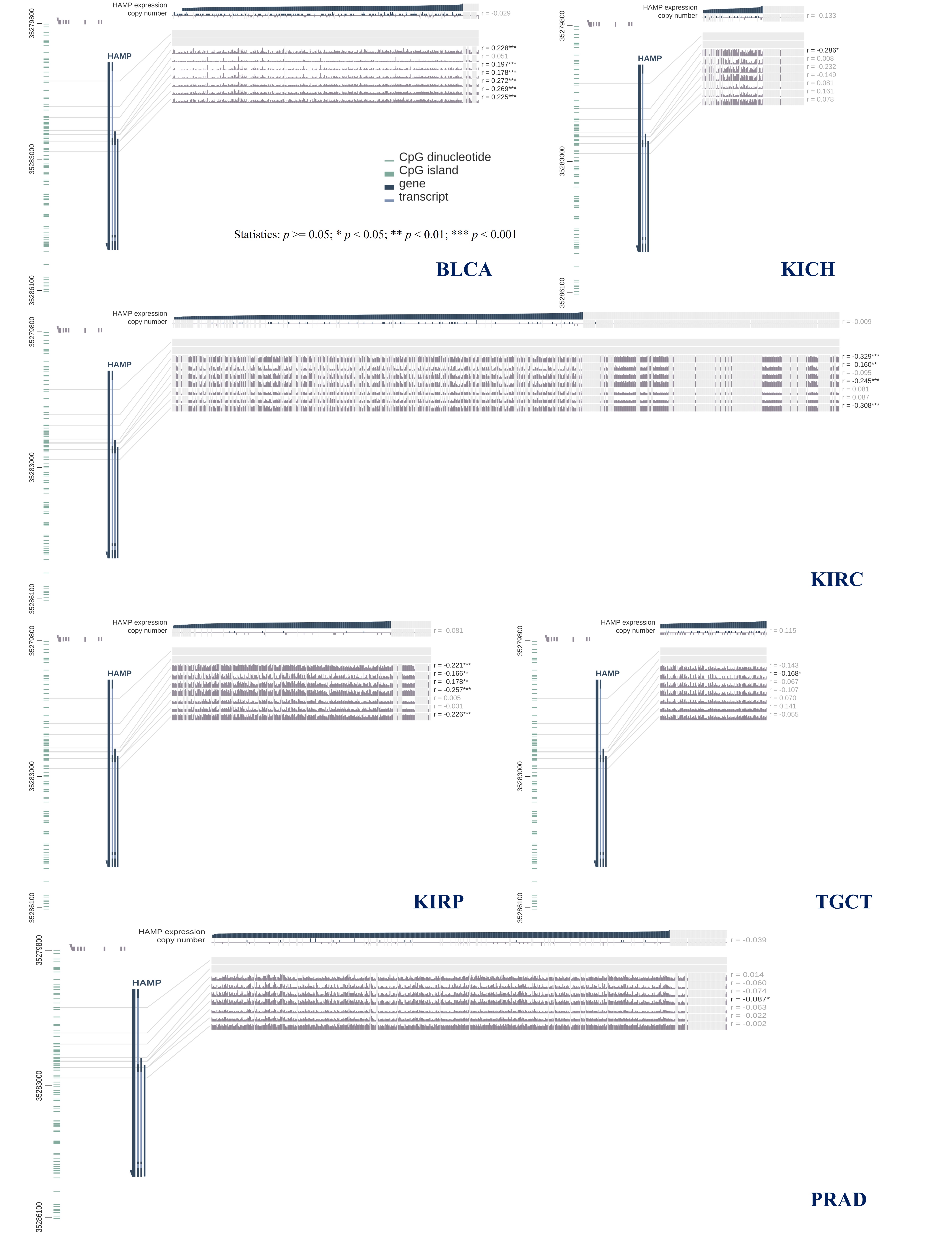

Supplement: Supplementary file 2 [file Image1.JPEG]
